# Molecular Weight-Dependent Oxidation and Optoelectronic Properties of Defect-Free Macrocyclic Poly(3-hexylthiophene)

**DOI:** 10.3390/polym15030666

**Published:** 2023-01-28

**Authors:** Ryohei Sato, Atsuo Utagawa, Koji Fushimi, Feng Li, Takuya Isono, Kenji Tajima, Toshifumi Satoh, Shin-ichiro Sato, Hiroshi Hirata, Yoshihiro Kikkawa, Takuya Yamamoto

**Affiliations:** 1 Graduate School of Chemical Sciences and Engineering, Hokkaido University, Sapporo 060-8628, Japan; 2 Division of Applied Chemistry, Faculty of Engineering, Hokkaido University, Sapporo 060-8628, Japan; 3 Division of Bioengineering and Bioinformatics, Faculty of Information Science and Technology, Hokkaido University, Sapporo 060-0814, Japan; 4 National Institute of Advanced Industrial Science and Technology (AIST), Tsukuba Central 5, 1-1-1 Higashi, Tsukuba 305-8565, Japan

**Keywords:** cyclic polymer, poly(3-hexylthiophene), molecular weight dependence, oxidation, polaron, biradical, dication, cyclic voltammetry, UV–Vis–NIR spectroscopy, ESR spectroscopy

## Abstract

The redox behaviors of macrocyclic molecules with an entirely π-conjugated system are of interest due to their unique optical, electronic, and magnetic properties. In this study, defect-free cyclic P3HT with a degree of polymerization (DP_n_) from 14 to 43 was synthesized based on our previously established method, and its unique redox behaviors arising from the cyclic topology were investigated. Cyclic voltammetry (CV) showed that the HOMO level of cyclic P3HT decreases from –4.86 eV (14 mer) to –4.89 eV (43 mer), in contrast to the linear counterparts increasing from –4.94 eV (14 mer) to –4.91 eV (43 mer). During the CV measurement, linear P3HT suffered from electro-oxidation at the chain ends, while cyclic P3HT was stable. ESR and UV–Vis–NIR spectroscopy suggested that cyclic P3HT has stronger dicationic properties due to the interactions between the polarons. On the other hand, linear P3HT showed characteristics of polaron pairs with multiple isolated polarons. Moreover, the dicationic properties of cyclic P3HT were more pronounced for the smaller macrocycles.

## 1. Introduction

Fully conjugated macrocyclic molecules are regarded as infinitely conjugated systems without effects from termini and have attracted considerable attention due to their unique optical, electronic, and magnetic properties resulting from their cyclic topology [1,2]. For example, compared to the corresponding linear counterparts, macrocyclic oligothiophenes have been reported to exhibit a blue shift in the maximum absorption wavelength and exciton delocalization due to planarization [3,4,5,6]. Recently, Kim et al. revealed ring size-dependent dynamics of structural relaxation in exciton-delocalized cyclic thiophenes [7]. Furthermore, Yamago et al. investigated the chemical properties of cycloparaphenylene (CPP) by introducing two radicals and found that unique properties such as in-plane aromaticity emerge depending on the ring size [8].

The redox behavior of conjugated polymers is very important for applications to optoelectronic devices because the conductivity of conjugated polymers can be increased from an insulator level to a conductor level by doping, and various physicochemical properties such as color can be changed. There are several theoretical and computational studies on the oxidation of cyclic macromolecules. Gidron et al. reported that the dodecameric cyclic oligothiophene has a distorted structure in the ground state, whereas the dihedral angle between neighboring thiophenes is almost zero in the radical cationic state, i.e., the macrocycle has a planar structure [9]. On the other hand, the two-electron oxidized form has a planar structure even in the more distorted 5–9 mers in the ground state. Yamago et al. experimentally reported complete charge delocalization in the oxidized state of [5]–[12]CPP from UV–Vis–NIR absorption and electron spin resonance (ESR) spectroscopy [10,11]. Moreover, in the two-electron oxidation state, they found that in the range of [5]–[8]CPP, the radicals fully interact with each other to form a bipolaron (dication) state, whereas in the range of [10]–[12]CPP, a partial polaron pair (biradical) state contributes strongly. Iyoda et al. reported that hexameric cyclic oligothiophene shows two sharp absorptions in the UV–Vis–NIR spectrum, which is typical for dications with closed-shell structures, while nonameric cyclic oligothiophene exhibits a broad absorption and has an open-shell biradical character [12]. They also found that the one-electron oxidized state of hexameric cyclic oligothiophene forms a dimer at low temperatures due to the intermolecular interactions, and it also exhibits Hückel-type antimagnetic ring current effects [13]. Bäuerle et al. similarly reported that decameric cyclic oligothiophene has a dicationic structure in the two-electron oxidized state and forms a dimer in the one-electron oxidized state [14].

**Figure 1:**
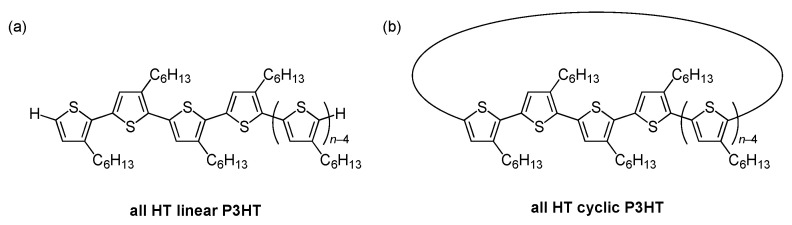
Chemical structures of (**a**) all head-to-tail linear P3HT and (**b**) all head-to-tail cyclic P3HT.

Previously, our research group achieved the synthesis of defect-free all head-to-tail cyclic poly(3-hexylthiophene) (P3HT) with a fully conjugated structure (Figure 1b) by applying the controlled synthesis of P3HT with an appropriate initiator and the homocoupling reaction of organostannane, which overcomes the conventional problems in the synthesis of conjugated macrocycles [15]. As a result of comparison with the corresponding linear counterpart (Figure 1a) and cyclic P3HT having a head-to-head bond as a defect in the regioregularity, the forbidden 0–0 transition in the fluorescence spectrum based on the selection rule established for cyclic aromatic hydrocarbons was observed only for the defect-free all head-to-tail cyclic P3HT [16]. Thus, we concluded that the ring topology and regioregularity have a significant effect on the optoelectronic properties. Although fully conjugated cyclic P3HT is a very interesting material, its structures and charge/spin states in the redox states have not been studied.

In this study, fully conjugated linear and cyclic P3HT ranging from a degree of polymerization (DP_n_) of 14 to 43 were synthesized based on our previously established method. Furthermore, the chemical oxidation was applied to them, and the effects of the cyclic topology and molecular weight on their optoelectronic properties were investigated. From cyclic voltammetry (CV), the HOMO level decreased as DP_n_ for cyclic P3HT, in contrast to the increasing HOMO level in linear P3HT in general. In addition, the cyclic P3HT was stable against the undesired intermolecular coupling reaction in response to electro-oxidation due to the absence of termini. ESR and UV–Vis–NIR spectral measurements indicated that cyclic P3HT has a larger contribution of dication and more delocalized charges than its linear counterparts.

## 2. Materials and Methods

### 2.1. Materials

Magnesium, turnings (Tokyo Chemical Industry Co., Ltd., Tokyo, Japan); 1,2-dibromoetane (>99.0%, Tokyo Chemical Industry Co., Ltd.); 2-bromo-3-hexylthiophene (>97.0%, Tokyo Chemical Industry Co., Ltd.); [1,3-bis(diphenylphosphino)propane]dichloronickel(II) (Ni(dppp)Cl_2_) (Sigma-Aldrich, St. Louis, MO, USA); 2-bromo-3-hexyl-5-iodothiophene (>97.0%, Tokyo Chemical Industry Co., Ltd.); isopropylmagnesium chloride–lithium chloride (*i*-PrMgCl·LiCl) (15% in tetrahydrofuran (THF), ca. 1 mol L^−1^, Tokyo Chemical Industry Co., Ltd.); boronic acid-functionalized resin (boronic acid, polymer-bound, 200–400 mesh, extent of labeling: 1.4–2.2 mmol/g loading, 1% cross-linked with divinylbenzene, Sigma-Aldrich); potassium carbonate (>80%, Wako Pure Chemical Industries, Ltd.) tetrakis(triphenylphosphine)palladium(0) (Pd(PPh_3_)_4_) (97.0%, Tokyo Chemical Industry Co., Ltd.); *N*,*N*,*N’*,*N’*-tetramethylethylenediamine (TMEDA) (>98.0%, Tokyo Chemical Industry Co., Ltd.); *sec*-butyllithium (*sec*-BuLi) (in cyclohexane, *n*-hexane, ca. 1 mol L^−1^, Kanto Chemical Co., Inc.); trimethyltin chloride (Me_3_SnCl) (>98.0%, Tokyo Chemical Industry Co., Ltd.); bis(benzonitrile)palladium(II) chloride (PdCl_2_(PhCN)_2_) (>95%, Sigma-Aldrich); triphenyl phosphine (PPh_3_) (>97.0%, Wako Pure Chemical Industries, Ltd.); chloroacetone (>95.0%, stabilized with magnesium oxide, Tokyo Chemical Industry Co., Ltd.); phosphoryl chloride (POCl_3_) (99.0%, Tokyo Chemical Industry Co., Ltd.); aminomethyl-functionalized resin (aminomethyl polystyrene resin cross-linked with 1% DVB (200–400 mesh) (2.0–3.0 mmol g^−1^)), 1,4-diazabicyclo[2.2.2]octane (DABCO) (>98.0%, Tokyo Chemical Industry Co., Ltd.); silver hexafluoroantimonate(V) (AgSbF_6_) (>97.0%, Tokyo Chemical Industry Co., Ltd.); 2,2,6,6-tetramethylpiperidine-1-oxyl (TEMPO) (98%, Angene Chemical, Nanjing, China); THF super dehydrated, stabilizer free (99.5%, Wako Pure Chemical Industries, Ltd., Osaka, Japan); *n*-hexane (>99.5%, Kanto Chemical Co. Ltd., Tokyo, Japan); acetone (>99.5%, Kanto Chemical Co. Ltd.); chloroform (CHCl_3_) (>99.0%, Kanto Chemical Co. Ltd.); methanol (MeOH) (>99.6%, Junsei Chemical Co. Ltd.); dichloromethane (CH_2_Cl_2_), specially prepared reagent for fluorometry (≥99.5%, NACALAI TESQUE, Co., Ltd., Kyoto, Japan); CDCl_3_ (99.8 atom%D, contains 0.5 wt.% silver foil as stabilizer, 0.03% (*v*/*v*) TMS, Sigma-Aldrich); dichloromethane-*d*_2_ (99.9 atom%D, Tokyo Chemical Industry Co., Ltd.) were used as received without purification. THF for cyclization (stabilizer free, water content <10 ppm, Kanto Chemical Co. Ltd.) and *N*,*N*-dimethylformamide (DMF), super dehydrated (99.5%, Kanto Chemical Co. Ltd.) were purified by a solvent purification system (MBRAUN MB-SPS-Compact). Toluene (>98.0%, Kanto Chemical Co. Ltd.) was distilled over Na. The polymerization experiments were carried out in an MBRAUN stainless steel glovebox equipped with a gas purification system (molecular sieves and copper catalyst) in a dry argon atmosphere (H_2_O, O_2_ <1 ppm). The moisture and oxygen contents in the glovebox were monitored by an MB-MO-SE 1 moisture sensor and an MB-OX-SE 1 oxygen sensor, respectively.

### 2.2. Nuclear Magnetic Resonance (NMR) Spectroscopy

^1^H NMR (400 MHz) spectra were measured in CDCl_3_ using a JEOL JNM-ECS400 instrument. Variable temperature ^1^H NMR (600 MHz) spectra were measured in CD_2_Cl_2_ using a JEOL JNM-ECZ600r instrument. Tetramethyl silane was used as a reference standard.

### 2.3. Analytical Size Exclusion Chromatography (SEC)

Analytical SEC was conducted on a Shodex GPC-101 gel permeation chromatography system (Shodex DU-2130 dual pump, Shodex RI-71 reflective index detector, and Shodex ERC-3125SN degasser) equipped with a Shodex KF-G guard column (4.6 mm × 10 mm; pore size, 8 μm) and two Shodex KF-804L columns (8 mm × 300 mm) in series. Polystyrene standard samples were used for calibration, and THF was used as an eluent at a flow rate of 1.0 mL min^–1^ at 40 °C. The molecular weights of P3HT were estimated by the following equation: *M*_n,SEC_(P3HT) = *M*_n,SEC_(PS)/1.67 [17]. All the *M*_n_ values presented in the study were determined by SEC.

### 2.4. Preparative SEC

A Japan Analytical Industry Model LC-9201 recycling preparative HPLC system (RI-50s detector and PI-50 pump) equipped with JAIGEL-2H and 3H columns in series was used. Traces were recorded with a JASCO 807-IT integrator. CHCl_3_ was used as a solvent, and the flow rate was set at 3.5 mL min^–1^.

### 2.5. Matrix-Assisted Laser Desorption Ionization Time-of-Flight (MALDI-TOF) Mass Spectrometry

MALDI-TOF mass spectra were measured on an AB SCIEX TOF/TOF 5800 system equipped with an OptiBeam On-Axis Laser in a reflector positive mode. The P3HT samples (in THF; 1.5 mg mL^–1^, 10 μL) were mixed with dithranol (in THF; 40 mg mL^–1^, 25 μL). The detected ionic species was obtained by the removal of an electron from the P3HT products; thus, the observed *m*/*z* values were equal to the molecular weight.

### 2.6. UV–Vis and UV–Vis–NIR Spectroscopies

UV–Vis and UV–Vis–NIR absorption spectra were obtained on a JASCO Ubset V-670 spectrophotometer at ambient temperature. The concentration of P3HT was 10 μg mL^–1^ in CHCl_3_ for the UV–Vis absorption spectra and 16.6 μg mL^−1^ in CH_2_Cl_2_ for the UV–Vis–NIR absorption spectra.

### 2.7. Raman Spectroscopy

Raman spectroscopy was performed on a RENISHAW in Via Reflex (*λ*_ex_ = 785 nm). The concentration of P3HT was 0.03 mg mL^–1^ in CHCl_3_, and the measurement conditions were as follows: Intensity 10%, 10 s, Scan 50, Static mode, Center 1400 cm^–1^.

### 2.8. Scanning Tunneling Microscope (STM)

STM observations were performed by using a Nanoscope IIIa and Nanoscope 8 multimode SPM (Bruker, MA). The STM tip was prepared from a mechanical cut of a Pt–Ir (90:10) wire. The polymers were dissolved in 1,2,4-trichlorobenzene to prepare 0.01–0.03 mM solutions. The solutions were dropped onto a freshly cleaved HOPG surface (ZYB grade, Momentive Performance Materials, OH). Observations were carried out at the HOPG/solvent interface. All the STM images were low pass filtered by using SPIP software (Image Metrology, Denmark). The tunneling conditions are described in each figure caption.

### 2.9. Cyclic Voltammetry (CV)

CV measurements were carried out on a Biologic SP-150 potentiostat with a three-electrode cell at a scan rate of 20 mV s^−1^. A platinum disk (diameter of 6 mm), platinum wire, and saturated AgNO_3_/Ag electrode were used as working electrode, counter electrode, and reference electrode, respectively. The sample concentration was 0.1 mg mL^−1^, except for **L_43_** and **C_43_**, which were measured at 0.03 mg mL^−1^ due to their solubility. Tetrabutylammonium hexafluorophosphate (TBAPF_6_) (0.1 M) in CH_2_Cl_2_ was used as an electrolyte. Before measurement, the working electrode was rinsed with CH_2_Cl_2_, polished with a 1 μm polishing diamond and 0.05 μm polishing alumina, ultrasonically cleaned with distilled water, and dried. The potential was calibrated with ferrocene/ferrocenium (Fc/Fc^+^), and the HOMO energy levels were estimated by the following equation: $E_{\mathrm{HOMO}}\left(\mathrm{eV}\right)=-e\left[E_{\mathrm{onset}\;}^{\mathrm{ox}}+4.80\right]$, where $E_{\mathrm{onset}\;}^{\mathrm{ox}}$ is the oxidation onsets versus the half potential of Fc/Fc^+^ [18]. Measurements were performed for 10 cycles for linear P3HT and 5 cycles for cyclic P3HT at ambient temperature.

### 2.10. Spectroelectrochemistry

An ALS Co., Ltd. 013,510 SEC-C Thin Layer Quartz Glass Spectrochemical cell Kit (Pt) equipped with a platinum mesh working electrode (6 × 7 mm^2^, 80 mesh) and a platinum wire counter electrode was used. The optical path length was 1.0 mm. The sample solutions and reference electrode were the same as in CV. The measurements were performed in a cell with an optical path length of 1 mm by transmitting the probe light through the mesh electrode on a JASCO Ubset V-670 spectrophotometer at ambient temperature. After 30 s had elapsed since the start of voltage application, measurements were made nine times in succession over a period of 10 min. This procedure was performed at 0.4, 0.1, 0, and –0.1 V versus Fc/Fc^+^.

### 2.11. Preparation of Radical Cation (P3HT^•+^) and Dication (P3HT^2+^)

The radical cationic and dicationic species of linear and cyclic P3HT were generated by adding 0–7 and 0–10 equiv, respectively, of AgSbF_6_ in CH_2_Cl_2_ (0.15 mL) to solutions of P3HT (0.02 mg, 1 equiv) in CH_2_Cl_2_ (0.20 mL), under an argon atmosphere at ambient temperature. The equiv of AgSbF_6_ was calculated with respect to the P3HT macromolecules.

### 2.12. Electron Spin Resonance (ESR) Spectroscopy

ESR spectroscopy was performed on a Bruker EMXplus spectrometer at ambient temperature. The conditions of the ESR measurements were as follows: modulation frequency of 100 kHz, modulation amplitude of 2 G, microwave power of 1.0 mW, and microwave frequency of ~9.9 GHz. The spectra were scanned in the range of 3490–3550 G with a sweep time of ~121 s and 5 sweeps/spectrum accumulation.

### 2.13. Data Fitting of ESR Spectra

To eliminate the influence of spectral noises in the comparison of spin concentrations from the ESR spectra, the fitting of the ESR spectra was performed. To simplify the calculation, the data points were downsampled to 1/8, and the fitting was performed using the Voigt function, which is the convolution of Gaussian and Lorentz functions. By comparing the spectra obtained by the experiment with those obtained by the fitting, we confirmed the validity of the fitting results.

## 3. Results and Discussion

### 3.1. Synthesis

The polymers used in this study were synthesized according to our previous report [15]. First, linear P3HT (**L_n_**) was synthesized by Grignard metathesis (GRIM) polymerization using an initiator, in which the 2-position of thiophene was replaced with Ni(dppp)Cl by a metal exchange reaction. For the cyclization reaction, a Pd-catalyzed homocoupling reaction of aromatic tin compounds was applied, which is a direct coupling method between thiophene rings. In the preset synthesis, defect-free cyclic P3HT was constructed by introducing a trimethylstannyl group at both ends of linear P3HT and performing an intramolecular ring-closing reaction. The impurity with inhomogeneity in the regioregularity generated during the GRIM polymerization and the remaining linear species during cyclization were removed by reacting with resins containing appropriate functional groups. Finally, preparative size exclusion chromatography (SEC) was used to obtain highly pure cyclic P3HT. By carrying out this procedure with different initiator/monomer ratios in the GRIM polymerization, 14, 21, 26, 29 and 43-meric cyclic P3HT (namely **C_14_**, **C_21_**, **C_26_**, **C_29_**, and **C_43_**, respectively) were synthesized. The cyclic structure was confirmed by the disappearance of the signal from the chain ends in ^1^H NMR and a shift in the isotope distribution by *m*/*z* = 2 in MALDI-TOF MS measurements, due to the elimination of two hydrogen atoms from the chain ends (Figures S1–S10).

### 3.2. SEC

The analytical SEC results for linear and cyclic P3HT with different DP_n_ are shown in Table 1 and Figure S11. The resulting polymers corresponded to an intended DP_n_ of 14, 21, 26, 29, and 43, and the polydispersity was less than 1.2, achieving sufficient polymerization control for the evaluation of the molecular weight dependence. In addition, as the molecular weight increased, the shifts of the peak top molecular weight (*M*_p,SEC_) due to cyclization tended to be larger. For example, the *M*_p,SEC_ ratio of linear and cyclic P3HT (*M*_p,C_/*M*_p,L_) was 0.92 for 14-mer and 0.71 for 43-mer. Thus, the hydrodynamic volume reductions due to the cyclization became more remarkable as the molecular weight increased because the ideal ring structure was likely maintained in the small-sized cyclic P3HT, whereas the larger-sized cyclic P3HT probably distorted, and its cyclic conformation collapsed.

### 3.3. STM

**Figure 2:**
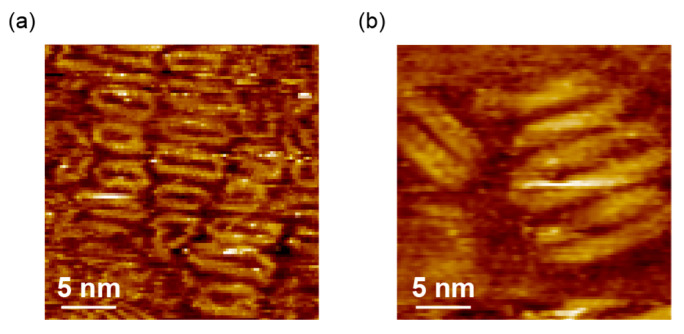
STM images of (**a**) **C_22_** (*I* = 50 pA, *V* = –498 mV) (**left**) and (**b**) **C_43_** (*I* = 39 pA, *V* = –907 mV) (**right**).

Observation by STM was performed to directly visualize **C_43_** and compare it to that of **C_22_**, which was previously reported [15]. The cyclic topology was clearly observed in **C_22_** (Figure 2 left)—however, it was not easy for **C_43_** (Figure 2 right). In the observation of **C_43_**, ring-like structures were observed, and these had approximately twice the circumference of **C_22_** (**C_22_**; 13 nm, **C_43_**; 23 nm). This result is one piece of evidence for the formation of macrocycles corresponding to the molecular weight observed by analytical SEC.

### 3.4. Raman Spectroscopy

Regarding cyclic oligo- or polythiophene and their derivatives, the calculation results of Bendikov et al. show that the conformation of cyclic oligothiophenes is energetically more stable if it has cisoid structures (Figure S12 left) for smaller than 14-mer and transoid structures (Figure S12 right) for larger than 16-mer [19]. Bäuerle et al. also showed that 10-meric cyclic oligothiophene has a cisoid structure [20]. Moreover, the calculation results suggest that the peaks corresponding to the C=C stretching vibration in the Raman spectrum of cyclic oligothiophenes are found at 1420–1460 cm^–1^ in the cisoid structures and 1485–1505 cm^–1^ in the transoid structures; thus, the conformational difference appears in a clear peak shift of approximately 50 cm^–1^ [19]. Therefore, we performed Raman spectroscopy for 14, 21, 26, and 43-mers of cyclic P3HT in CHCl_3_ to analyze the ring size-dependent cisoid/transoid structure. As a result, a peak corresponding to the C=C stretching vibration was observed at around 1476 cm^–1^ in all samples (Figure S13); the ring size-dependent difference was only about 3 cm^–1^. Additionally, the Raman shifts were almost equal to that of **L_21_**, which is known to have a transoid structure, suggesting that all cyclic P3HTs have a transoid structure, independent of the ring size.

### 3.5. UV–Vis Spectroscopy

**Figure 3:**
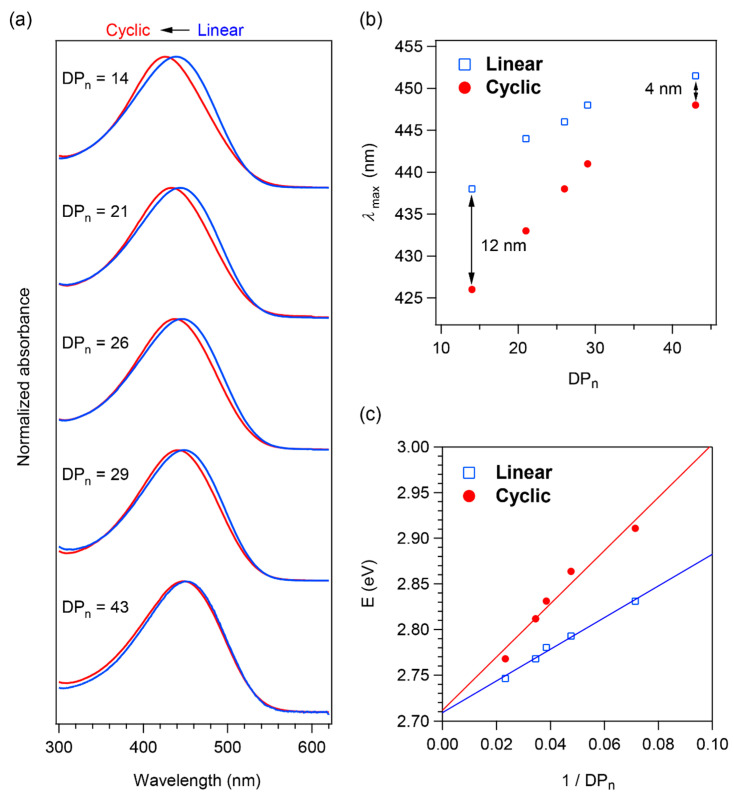
UV–Vis spectra of (**a**) linear (blue) and cyclic (red) P3HT with DP_n_ = 14, 21, 22, 26, and 43. (**b**) Maximum absorption wavelengths (Abs *λ*_max_) of linear (blue square) and cyclic (red circle) P3HT. (**c**) Plots of the transition energies determined from Abs *λ*_max_ against the reciprocal of DP_n_ of linear (blue square) and cyclic (red circle) P3HT.

UV–Vis absorption spectroscopy was performed for the solutions of linear and cyclic P3HT, in order to investigate the influences on the optical properties arising from the topologies. The wavelengths of the maximum absorption (Abs *λ*_max_) were 438 and 426 nm for **L_14_** and **C_14_**, respectively (Figure 3a, Table 2). Similarly, a blue shift due to cyclization was also observed in 21–43 mers, suggesting a decrease in the effective conjugation length upon cyclization. Moreover, with the increasing size of the macrocycles, the contribution of the cyclic topology on Abs *λ*_max_ tended to decrease, with the difference in Abs *λ*_max_ between linear and cyclic P3HT being 12 nm for the 14 mers and 4 nm for the 43 mers (Figure 3b). The blue shift in Abs *λ*_max_, which was prominent in the smaller ring sizes, was likely due to the magnitude of the curvature or ring strain [3]. Subsequently, Abs *λ*_max_ was plotted in energy units versus 1/DP_n_, and both linear and cyclic P3HT series were linearly fitted. By extrapolating these plots, the transition energy of infinitely long P3HT was calculated to be 2.70 eV (460 nm) for both linear and cyclic P3HT, which is reasonable because no ring strain would be expected in infinitely large cyclic P3HT. Moreover, the optical band gaps ($E_{g}^{\mathrm{opt}}$) were calculated from the absorption onset (Abs *λ*_onset_) of the S_0_ → S_2_ transition; the bandgap tended to decrease as DP_n_ increased (Figure S14 and Table 2) [20].

### 3.6. CV

**Figure 4:**
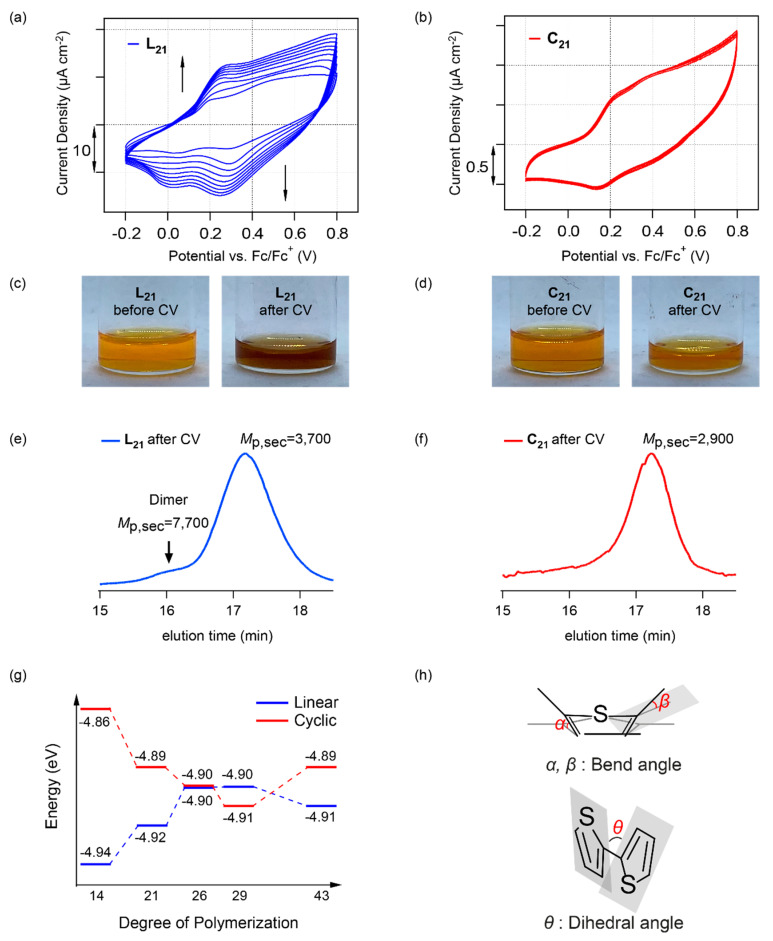
Cyclic voltammograms of (**a**) **L_21_** (2nd–10th cycles) and (**b**) **C_21_** (2nd–5th cycles). Photographs of (**c**) **L_21_** before (**left**) and after (**right**) CV, and (**d**) **C_21_** before (**left**) and after (**right**) CV. Analytical SEC traces of (**e**) **L_21_** and (**f**) **C_21_** after CV. (**g**) Energy diagrams of HOMO levels for linear (blue) and cyclic (red) P3HT. (**h**) Schematic illustrations of the bend angles (**above**) and dihedral angle (**below**) in polythiophene.

**Scheme 1:**
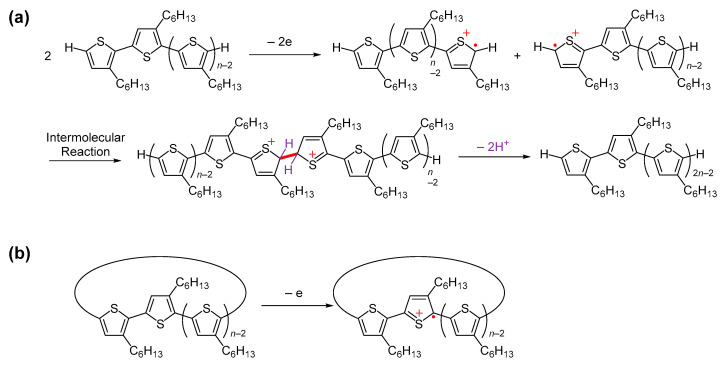
Schemes of electrochemical oxidation and potentially associated intermolecular reactions for (**a**) linear and (**b**) cyclic P3HT. The intermolecular reaction of linear P3HT occurs through subsequent coupling of the terminal radicals and deprotonation. The intermolecular reaction can be in a head-to-head, head-to-tail, or tail-to-tail manner. On the other hand, cyclic P3HT maintains its structure due to the lack of termini.

CV measurements are widely used to determine HOMO levels. The cyclic voltammograms of linear polythiophenes have been studied extensively, and in the process of the measurement, electropolymerization and/or interpolymer reactions are known to proceed, resulting in precipitation on the electrode and an increase in redox current density [21,22]. On the other hand, cyclic polymers are expected to be electrochemically stable because there are no chain ends to undergo such reactions. Thus, we performed CV measurements to compare the redox behaviors of linear and cyclic P3HT. An increase in both oxidation and reduction currents was observed in **L_21_** as the cycles proceeded, and the measurement was ended at –0.2 V versus Fc/Fc^+^. The second to tenth cycles of the cyclic voltammogram are shown in Figure 4a. Black–purple aggregates were observed near the electrodes after the measurement (Figure 4c). This result means that the main chain ends of the linear polymers are oxidized to generate radical cation species, which react to other polymer chains to form intermolecularly coupled products (Scheme 1a). This scheme is presumed to be the same as the mechanism of the electropolymerization of 3-hexylthiophene [21,23,24], and the intermolecular reaction was confirmed by the appearance of a shoulder peak corresponding to **L_21_** dimers in the analytical SEC trace after the CV measurement (Figure 4e). On the other hand, **C_21_** gave a stable cyclic voltammogram without an increase in the current density, and the second to fifth cycles of the cyclic voltammogram are shown in Figure 4b. No change in the color or dimer formation was observed in the analytical SEC trace after the CV measurement (Figure 4d,f, Scheme 1b). Hence, the electrochemical stability of cyclic P3HT in the solution state was suggested. Additionally, the current density in the cyclic P3HT was much smaller than that in the linear P3HT, where the adsorption of polythiophene to the electrode causes an increase in the current value [23]. Therefore, we conclude that the small current density for cyclic P3HT is due to the lack of chain ends in the cyclic species, which prevent the intermolecular reaction and adsorption on the electrode.

Next, the voltammograms of cyclic P3HT with various DP_n_ were compared (Figure S15). Again, the linear P3HT did not give stable cyclic voltammograms due to the electrochemical reaction, while the cyclic P3HT exhibited constant voltammograms with a similar shape for all DP_n_. The onset potential of the first oxidation ($E_{\mathrm{onset}}^{\mathrm{ox}}$) of each polymer was used to determine the HOMO level (Table 2). The HOMO levels of linear P3HT were –4.94 eV for **L_14_** and –4.91 eV for **L_43_**, showing general characteristics of a conductive polymer with a slight increase in the HOMO level as DP_n_ (Figure 4g). On the other hand, for cyclic P3HT, the HOMO level slightly decreased with increasing DP_n_, from –4.86 eV for **C_14_** to –4.91 eV for **C_43_**. Such a tendency of a decreasing HOMO level with increasing DP_n_ was also reported for [*n*]cycloparaphenylenes [25,26]. The previous experimental and theoretical studies show that as the size of a π-conjugated macrocycle increases, the bending of each aromatic ring unit decreases, and the dihedral angle between neighboring aromatic ring units becomes larger [27]. Therefore, the causes for the decrease in the HOMO level in cyclic P3HT likely arose from the bending and torsion effects (Figure 4h).

### 3.7. Spectroelectrochemistry

**Figure 5:**
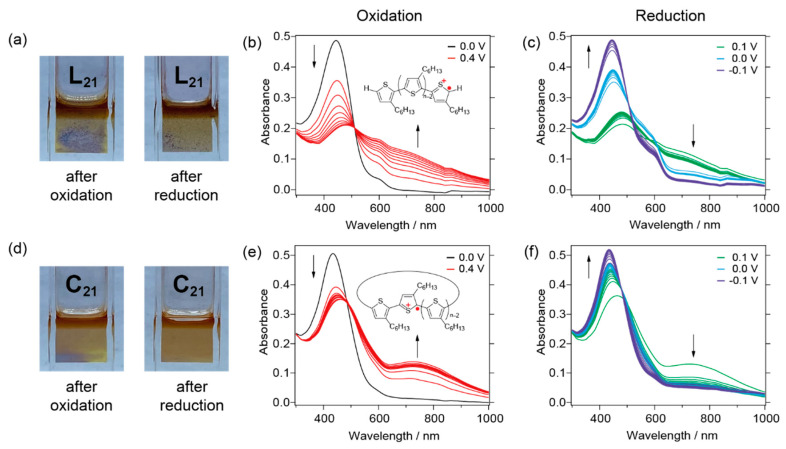
Photographs of (**a**) **L_21_** and (**d**) **C_21_** in spectroelectrochemistry measurements, and UV–Vis–NIR spectra of (**b**) **L_21_** and (**c**) **C_21_** during oxidation at 0.4 V, and of (**e**) **L_21_** and (**f**) **C_21_** during reduction at 0.1, 0.0, and –0.1 V.

To further analyze the redox behavior of **L_21_** and **C_21_**, UV–Vis absorption spectra were measured during electrochemical oxidation and reduction. A voltage of 0.4 V vs. Fc/Fc^+^ was applied onto a polymer solution to oxidize P3HT for 10 min, during which UV–Vis absorption spectra were recorded every minute. Following the oxidation, the applied voltage was lowered to 0.1 V and held constant for 10 min. Successively, this procedure was performed at 0 and –0.1 V. The time course absorption spectra for **L_21_** and **C_21_** at 0.4 V revealed a diminution of the intensity at Abs *λ*_max_ (444 nm), while absorption bands appeared at around *λ* = 750 nm for both **L_21_** and **C_21_** (Figure 5b,e). The intensity at 750 nm increased over time during oxidation, and upon reduction at 0.1, 0, and –0.1 V, these peaks gradually decreased. (Figure 5c,f). This suggests that the redox behavior observed in the cyclic voltammogram is due to the generation and reversion of a one-electron oxidized state (polaron). Since the second oxidation of P3HT proceeds at around 0.7 V vs. Fc/Fc^+^ [28], no dication should have been produced by the present experiment. These results are in good agreement with those reported by the groups of Bäuerle and Iyoda for macrocyclic thiophene derivatives (polaron absorption bands at around *λ* = 500 and 700 nm) [12,14]. Furthermore, a shoulder peak observed at around 600 nm for **L_21_** was attributed to the vibronic absorption band from the π–π stacking of P3HT chains caused by aggregation [29]. In addition, the visible formation of black–purple precipitates suggests deposition of the intermolecularly reacted linear species (Figure 5a). These results indicate that the prominent decrease in Abs *λ*_max_ for **L_21_** is due to the decrease in the polymer concentration by the precipitation of the intermolecularly reacted products. On the other hand, the absorption peak of the one-electron oxidation band (*λ* = 750 nm) clearly appeared in **C_21_** because the chain ends did not exist due to its unique cyclic topology; thus, the generated polarons stably persisted. Moreover, no purple precipitation due to the intermolecular reaction was observed in the photographs after redox (Figure 5d). Comparing the absorption spectra before and after the redox reactions, both **L_21_** and **C_21_** showed a slight increase in the baseline. This was attributed to absorption from the residues of the polaron because diffusion is the rate-limiting factor in this system [30].

### 3.8. UV–Vis–NIR Spectroscopy of Chemically Oxidized P3HT

**Figure 6:**
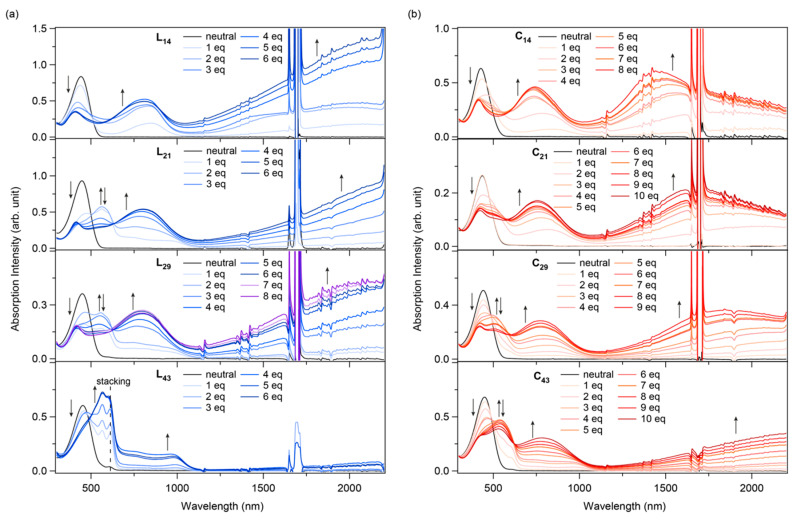
UV–Vis–NIR spectra of oxidized (**a**) linear and (**b**) cyclic P3HT with a DP_n_ of 14–43. “eq” indicates the molar equivalent of added AgSbF_6_ with respect to the P3HT macromolecules.

For cyclic thiophenes of a large size, the investigation of photoelectronic properties in the chemically oxidized state has rarely been performed. In order to investigate the effects of the topology and molecular weight on the optoelectronic properties of P3HT in the oxidized states, UV–Vis–NIR absorption spectra were monitored upon chemical oxidation. Oxidized P3HT in CH_2_Cl_2_ were prepared by adding 1–10 equiv of AgSbF_6_ with respect to the P3HT macromolecules. As a result, oxidized linear P3HT showed new absorption bands at longer wavelengths for all DP_n_ (Figure 6a, Table 3). For example, neutral **L_14_** showed a strong absorption band at *λ*_max_ = 438 nm, whereas multiply oxidized **L_14_** with 6 equiv of AgSbF_6_ showed two new absorption bands at *λ*_max_ = 820 nm and *λ*_max_ > 2200 nm, which were also observed in **L_21_** and **L_29_**. In the case of **L_43_**, the absorption due to aggregation appeared strongly at 613 nm because it was not fully dissolved, and the absorption due to the oxidation state only partly appeared.

Similarly, for cyclic P3HT, new absorption bands at longer wavelengths were observed in the oxidized states compared to the neutral state (Figure 6b, Table 3). For instance, neutral **C_14_** showed a strong absorption band at *λ*_max_ = 428 nm, whereas for multiply oxidized **C_14_** with 10 equiv of AgSbF_6_, absorption bands were observed at *λ*_max_ = 740 and 1440 nm. This was also found for **C_21_**, **C_29_**, and **C_43_**. Moreover, a ring size dependence for these absorptions can be seen. Specifically, **C_21_** and **C_29_** had *λ*_max_ at around 1700 and 1840 nm, respectively, and that exceeded 2200 nm for **C_43_**.

Zozoulenko et al. studied the UV–infrared transition in p-doped P3HT experimentally by absorption spectroscopy and computationally by density functional theory (DFT) and tight-binding DFT [31]. The absorption at 430–450 nm attributes the transition from the valence band to the conduction band in the neutral state (denoted as *T*_N_). Three new absorption bands were also observed by oxidation: one at 0.4–0.6 eV (2070–3100 nm) due to excitation from the valence band to the spin-resolved orbitals located in the interband gap (denoted as *T*_P/B_); another at 2.1–2.4 eV (520–590 nm) due to a transition from the valence band to the conduction band (denoted as *T*_C_); and the third absorption band at 1.2–1.5 eV (830–1030 nm) attributed the superposition of *T*_P/B_ and *T*_C_ (denoted as *T*_P/B+C_). Similar calculations have also been reported for CPP, poly(3,4-ethylenedioxythiophene) (PEDOT), and other π-conjugated polymers [11,32,33]. These results suggest that the absorption in Figure 6 at 500–570 nm is for *T*_C_, the one at 740–820 nm is for *T*_P/B+C_, and the last one observed at >1440 nm is for *T*_P/B_.

Moreover, previous reports have shown that cyclo[10]thiophene with delocalized polarons have sharp absorption bands of nearly equal intensities at around 720 nm and 1260–1280 nm, and delocalized bipolarons have one strong absorption band at around 850 nm [14,34]. On the other hand, oligothiophenes with a DP_n_ of approximately 10 with a localized polaron have *λ*_max_ at around 840 nm and 2050 nm. In the case of bipolarons, the 2050 nm absorption band blue-shifts to around 1700 nm and has two absorption bands with different intensities [14,35]. In addition, Iyoda et al. showed that hexameric cyclic oligothiophene has sharp absorption bands of nearly equal intensities in the bipolaron state, while Bäuerle et al. also reported that dodecameric cyclic oligothiophene shows two equally sharp absorption bands in the bipolaron state (Iyoda et al., 600 nm and 1200 nm; Bäuerle et al., 683 nm and 1334 nm) [13,14]. Furthermore, Yamago et al. reported that the two-electron oxidized state of CPPs with a DP_n_ of 5–8 has two equally sharp absorption bands, while that with a DP_n_ of 10–12 has two absorption bands with different intensities and bandwidths [11]. These results suggest that the two equally strong absorption bands appearing at around 742 nm and 1440 nm in **C_14_** are likely due to the high degree of symmetry arising from the charge-delocalized structure, as in the previous reports [13,14,34]. On the other hand, for cyclic oligothiophenes with a large ring size as well as linear oligothiophenes, the asymmetry of the macromolecule in the oxidized state is expected to lead to unequal intensities and bandwidths resulting from the charge-localized structure.

### 3.9. ESR Spectroscopy

**Figure 7:**
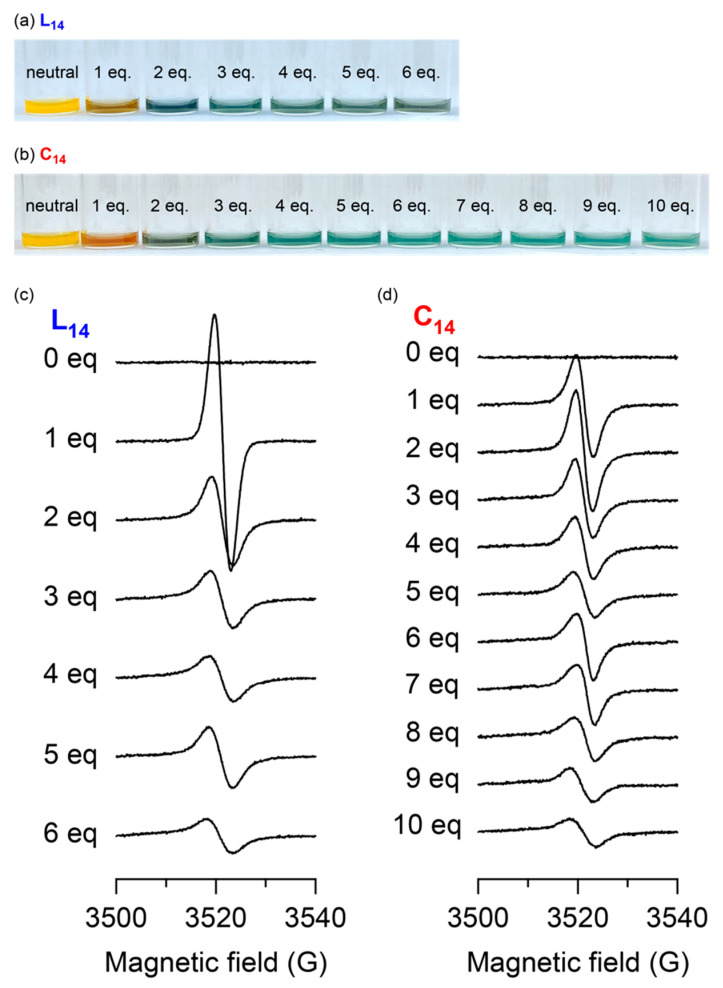
Photographs of (**a**) **L_14_** and (**b**) **C_14_** upon oxidation by AgSbF_6_. ESR spectra of (**c**) **L_14_** and (**d**) **C_14_** upon oxidation by AgSbF_6_. “eq” indicates the molar equivalent of added AgSbF_6_ with respect to the P3HT macromolecules.

**Figure 8:**
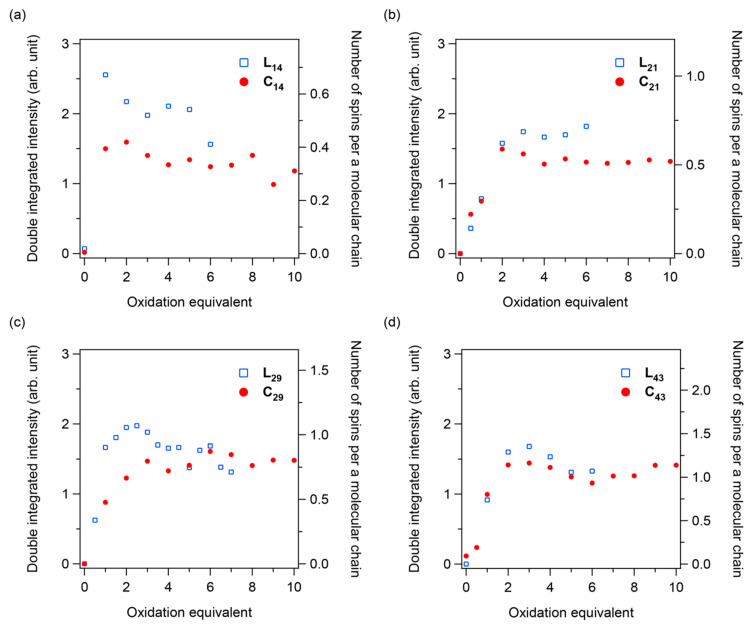
The double-integrated intensities of the first-derivative ESR spectra (left axis) and the number of spins per polymer chain (right axis) for a DP_n_ of (**a**) 14, (**b**) 21, (**c**) 29, and (**d**) 43 plotted against oxidant equiv. TEMPO was used as a standard sample to calculate the number of spins per polymer chain.

To further investigate the electronic properties upon oxidation, ESR measurements were performed on chemically oxidized linear and cyclic P3HT with AgSbF_6_. The **L_14_** solution, which was orange in the neutral state, became reddish brown upon the addition of ~1 equiv of AgSbF_6_ and green upon further addition (~6 equiv of AgSbF_6_) (Figure 7a). The **C_14_** solution also turned reddish brown upon the addition of ~1 equiv of AgSbF_6_ and green upon further addition (~10 equiv) (Figure 7b). Likewise, similar color changes were observed for P3HT with other DP_n_ (Figures S16–S18). In the ESR measurement of **L_14_**, the signal intensity was maximum at 1 equiv of the oxidant (Figure 7c). The signal intensity then decreased with the addition of 2 equiv or more of the oxidant, and broadening of the signal was observed. The linewidth of the spectrum depends on the strength of the interaction between the spin system and its surroundings [36]. In other words, the decrease in the intensity and broadening were due to an increase in the concentration of polarons and the interaction between the radicals. Figure 8 shows the double-integrated intensity of the first-derivative ESR spectrum, which is proportional to the spin concentration (number of unpaired electrons). This suggests that with up to 1 equiv of the oxidant, the number of unpaired electrons increases due to the formation of polarons on the main chain, while at the higher oxidant contents, the amount of paramagnetic charge carriers decreases due to the interaction between the generated polarons. This result is in good agreement with previous reports [37]. Next, for **C_14_**, the signal intensity reached its maximum at 2 equiv, and broadening of the linewidth was observed at higher equiv (Figure 7d). Thus, the calculated spin concentration in **C_14_** reached a maximum at 2 equiv of the oxidant and remained nearly constant thereafter (Figure 8a). Furthermore, the spin concentration of **C_14_** at the maximum was lower than that of **L_14_**, suggesting that the spin–spin interaction is larger in **C_14_** than in **L_14_**, i.e., the contribution of the dication is larger. The same trend was observed for P3HT with a DP_n_ of 21 and 29 (Figure 8b,c, Figures S16 and S17), with the difference between the linear and cyclic being more pronounced for smaller DP_n_. On the other hand, almost no difference was observed between linear and cyclic P3HT with a DP_n_ of 43 (Figure 8d, Figure S18). These suggest that smaller DP_n_ results in more restricted conformations for the macrocycle and a smaller dihedral angle between neighboring thiophene units, leading to a stronger interaction of polarons existing on the polymer chain [27].

Subsequently, the number of spins per polymer chain based on DP_n_ was calculated for cyclic P3HT (Figure 8). The spin concentration was determined by referring to that of 2,2,6,6-tetramethylpiperidine-1-oxyl (TEMPO) radical. For **C_14_**, the number of spins per polymer chain was 0.3–0.4 for between 1 and 8 equiv of the oxidant. On the other hand, that was nearly constant at 0.5, 0.75, and 1.0 for **C_21_**, **C_29_**, and **C_43_**, respectively, above 2–3 equiv of the oxidant. These results indicate that the number of spins per polymer chain increases with DP_n_ for cyclic P3HT, and one oxidation takes place for every 4–7 units of the thiophene units. This result is consistent with previous studies [32,37].

### 3.10. VT ^1^H NMR Spectroscopy

In the ESR spectral measurements, a decrease in spin density for several samples was observed with the addition of the oxidant, suggesting an interaction between spins in the multiply oxidized state. Furthermore, in a previous study, the temperature dependence of the transition between a polaron pair and bipolaron was reported [11]. In order to obtain more details on the cationic character of P3HT in the oxidized states, we performed variable temperature ^1^H NMR (VT ^1^H NMR) spectroscopy. Thus, 2 equiv of AgSbF_6_ was added to **L_14_**, and ^1^H NMR spectra were measured during stepwise cooling from 293 to 230 K (Figure S19). As a result, the signals derived from the proton at the 4-position of thiophene (*e*) and the methylene protons directly bonding to the 3-position of thiophene (*d*) were not observed due to the radical existing on the main chain. After the further addition of 4 more equiv of AgSbF_6_, the signals of *d* and *e* remained absent (Figure S20). Moreover, no change in the spectra due to temperature modulation was seen. We also performed the same procedure for **C_14_**, but the *d* and *e* signals were absent, and no temperature dependence was observed (Figures S21 and S22). These results indicate that linear and cyclic P3HT with a DP_n_ of 14–43 in the oxidized state have radical characters on the main chain at the temperatures, preventing the appearance of the NMR signals.

Considering the results of both ESR and UV–Vis–NIR spectra, it is suggested that linear P3HT adopts the state of polaron pairs in which isolated multiple polarons exist on the main chain. On the other hand, cyclic P3HT, especially with a small size, exhibited dicationic characters due to the interaction between polarons. In addition, the molecular weight dependence of the dicationic/biradical characters of cyclic P3HT was also clarified: the dicationic characters were stronger for smaller cyclic P3HT, and the biradical characters became dominant for lager cyclic P3HT, as well as for linear P3HT.

## 4. Conclusions

In this study, we synthesized fully conjugated defect-free cyclic P3HT with various DP_n_ (14–43mer) based on a polymeric approach and investigated its cyclic topology and molecular weight dependence. The absorption spectra showed a blue shift associated with cyclization, which was more pronounced for smaller DP_n_. In the cyclic voltammograms, the HOMO levels of linear P3HT increased with increasing DP_n_, whereas that of cyclic P3HT decreased due to the reduction in the bending of individual thiophene rings and the increase in the dihedral angle caused by the macrocyclic structure. Furthermore, while linear P3HT underwent intermolecular reactions by electro-oxidation, cyclic P3HT was stable against such reactions and retained its chemical structures due to the absence of the chain ends. ESR and UV–Vis–NIR spectroscopies showed that small cyclic P3HT has a large dicationic contribution and displays stronger charge delocalization compared to its linear counterparts. This study shows the control of the optoelectronic properties of π-conjugated polymers through their topology, which, when combined with chemical modification, would enable a wider range of property control.

## Figures and Tables

**Table 1:** Analytical SEC data for linear and cyclic P3HT with various DP_n_.

	*M*_n,SEC_ (g·mol^–1^) *^a^*	*M*_p,SEC_ (g·mol^–1^) *^a^*	*M*_w_/*M*_n_	*M*_p,C_/*M*_p,L_
**L_14_**	2300	2400	1.07	0.92
**C_14_**	2200	2200	1.03	
**L_21_**	3500	3500	1.05	0.83
**C_21_**	2900	2900	1.04	
**L_26_**	4300	4100	1.08	0.76
**C_26_**	3100	3100	1.05	
**L_29_**	5100	5300	1.17	0.68
**C_29_**	3900	3600	1.08	
**L_43_**	7200	8700	1.13	0.71
**C_43_**	5700	6200	1.19	

*^a^* The molecular weights of P3HT were estimated using the following equation: *M*_n,SEC_(P3HT) = *M*_n,SEC_(PS)/1.67.

**Table 2:** Optical and redox data for linear and cyclic P3HT with various DP_n_.

	Abs *λ*_max_ (nm) *^a^*	Abs *λ*_onset_ (nm) *^a,b^*	$E_{g}^{\mathrm{opt}}$ (eV) ^*b*^	$E_{\mathrm{onset}}^{\mathrm{ox}}$ (V) ^*c*^	HOMO (eV) *^d^*
**L_14_**	438	527	2.35	0.14	–4.94
**C_14_**	426	527	2.35	0.06	–4.86
**L_21_**	444	531	2.33	0.12	–4.92
**C_21_**	433	528	2.35	0.09	–4.89
**L_26_**	446	534	2.32	0.10	–4.90
**C_26_**	438	530	2.34	0.10	–4.90
**L_29_**	448	534	2.32	0.10	–4.90
**C_29_**	441	533	2.33	0.11	–4.91
**L_43_**	452	537	2.31	0.07	–4.91
**C_43_**	448	538	2.30	0.12	–4.89

*^a^* Measured in CHCl_3_. *^b^* Calculated from the S_0_ → S_2_ absorption band. *^c^* First oxidation potential in CH_2_Cl_2_/0.1 M TBAPF_6_ at 298 K, *v* = 20 mVs^–1^, potential versus ferrocene/ferrocenium (Fc/Fc^+^) couple. *^d^* HOMO energy levels were estimated using the following equation: $E_{\mathrm{HOMO}}\left(\mathrm{eV}\right)=-e\left[E_{\mathrm{onset}}^{\mathrm{ox}}+4.80\right]$, in which $E_{\mathrm{onset}}^{\mathrm{ox}}$ is oxidation onsets versus the half potential of Fc/Fc^+^ in the CV curves.

**Table 3:** Photophysical properties of linear and cyclic P3HT with various DP_n_.

	Transition (nm)			
	*T*_N_^*a*^	*T*_C_ *^b^*	*T*_P/B + C_ *^c^*	*T*_P/B_ *^d^*
**L_14_**	442	-	815	>2200
**C_14_**	428	-	742	1440
**L_21_**	447	562	766	>2200
**C_21_**	437	about 500	755	about 1700
**L_29_**	450	551	797	>2200
**C_29_**	442	508	773	1850
**L_43_**	453	566	-	-
**C_43_**	449	532	782	>2200

*^a^* Transition from the valence band to the conduction band in the neutral state. *^b^* Transition from the valence band to the conduction band in the oxidized states. *^c^* Superposition of *T*_P/B_ and *T*_C_. *^d^* Transition from the valence band to the spin-resolved orbitals located in the interband gap.

## Data Availability

The data presented in this study are available on request from the corresponding author.

## Supplementary Materials

The following supporting information can be downloaded at: https://www.mdpi.com/article/10.3390/polym15030666/s1, Figures S1–S5: ^1^H NMR spectra; Figures S6–S10: MALDI-TOF mass spectra; Figure S11: analytical SEC traces; Figure S12: transoid and cisoid structures of cyclic P3HT; Figure S13: Raman spectra; Figure S14: plots for optical band gaps versus DP_n_; Figure S15: cyclic voltammograms; Figures S16–S18: photographs and ESR spectra upon oxidation; Figures S19–S22: VT ^1^H NMR spectra.
