# Supplementary material for: Molecular Weight-Dependent Oxidation and Optoelectronic Properties of Defect-Free Macrocyclic Poly(3-hexylthiophene)
Source: Polymers (Basel). 2023 Jan 28;15(3):666. doi: 10.3390/polym15030666 (PMC9920727; doi:10.3390/polym15030666)
Supplement: Supplementary file 1 [file polymers-15-00666-s001.zip › polymers-2163488-supplementary.pdf]

Supplementary Materials

Molecular Weight-Dependent Oxidation and Optoelectronic Properties of Defect-Free Macrocyclic Poly(3-hexylthiophene)

Ryohei Sato,[1] Atsuo Utagawa,[1] Koji Fushimi,[2] Feng Li,[2] Takuya Isono,[2] Kenji Tajima,[2] Toshifumi Satoh,[2] Shin-ichiro Sato,[2] Hiroshi Hirata,[3] Yoshihiro Kikkawa,[4] and Takuya Yamamoto[2,*]

[1] Graduate School of Chemical Sciences and Engineering, Hokkaido University, Sapporo, Hokkaido 060–8628, Japan

[2] Division of Applied Chemistry, Faculty of Engineering, Hokkaido University, Sapporo, Hokkaido 060–8628, Japan

[3] Division of Bioengineering and Bioinformatics, Faculty of Information Science and Technology, Hokkaido University, Sapporo, Hokkaido 060–0814, Japan

[4] National Institute of Advanced Industrial Science and Technology (AIST), Tsukuba Central 5, 1–1–1 Higashi, Tsukuba, Ibaraki 305–8565, Japan

[*] Author to whom correspondence should be addressed.

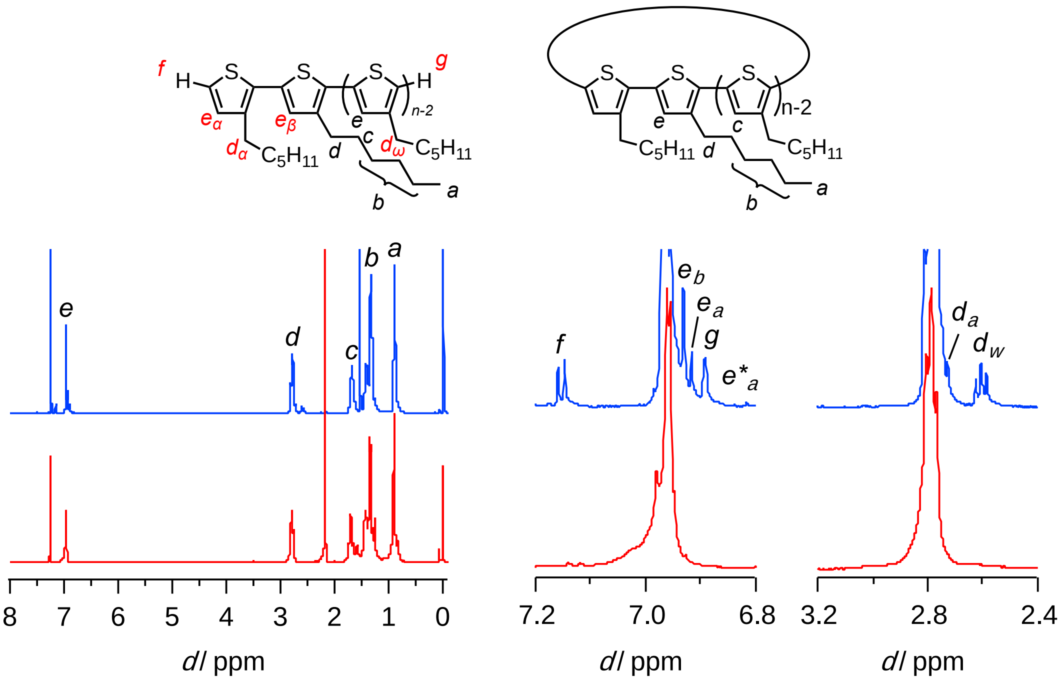

Figure S1. ^{1}H NMR spectra of $\mathbf{L_{14}}$ (blue) and $\mathbf{C_{14}}$ (red).

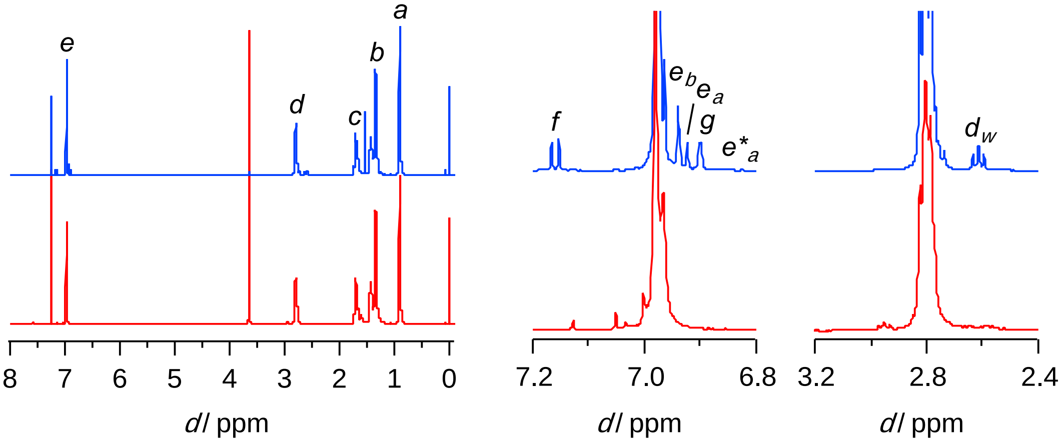

Figure S2. ^{1}H NMR spectra of $\mathbf{L_{21}}$ (blue) and $\mathbf{C_{21}}$ (red).

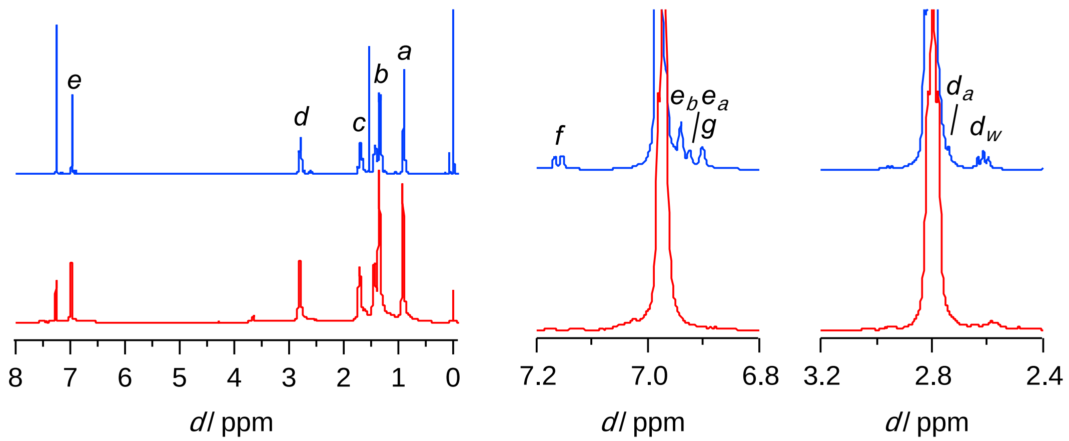

Figure S3. ^{1}H NMR spectra of $\mathbf{L_{26}}$ (blue) and $\mathbf{C_{26}}$ (red).

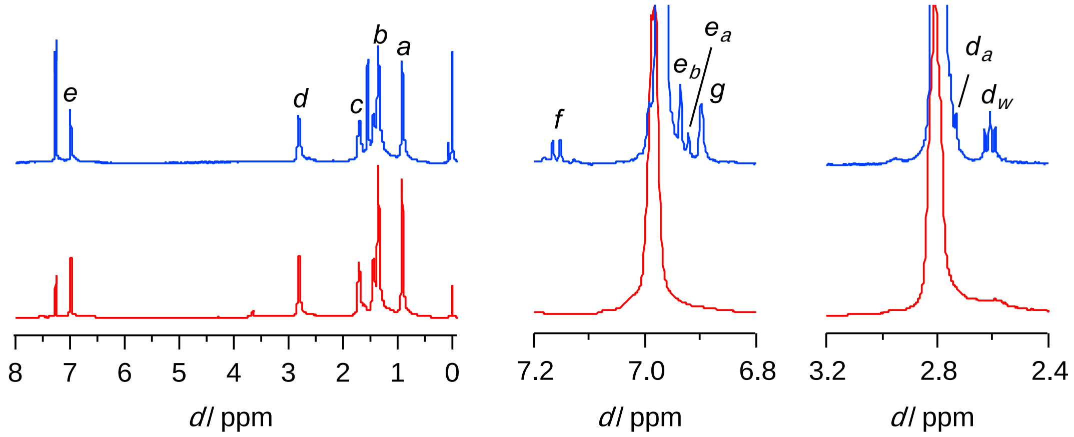

Figure S4. ^{1}H NMR spectra of $\mathbf{L_{29}}$ (blue) and $\mathbf{C_{29}}$ (red).

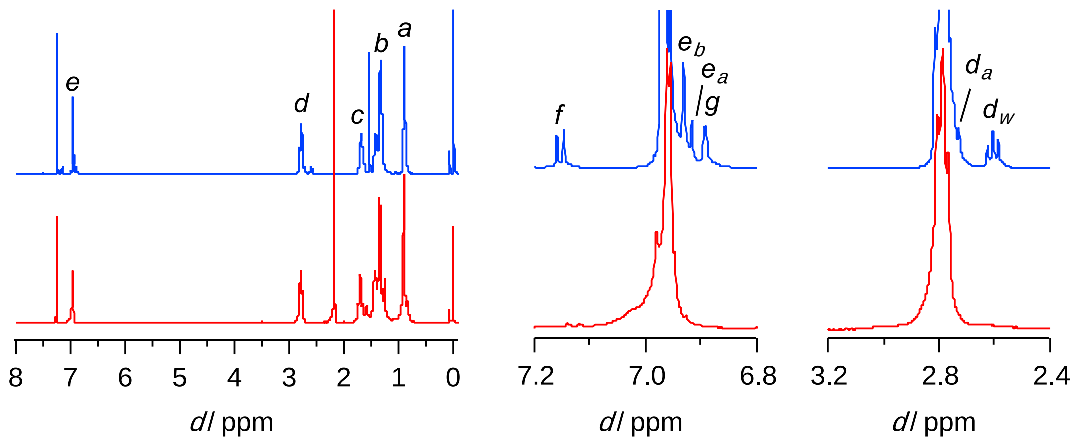

Figure S5. ^{1}H NMR spectra of $\mathbf{L_{43}}$ (blue) and $\mathbf{C_{43}}$ (red).

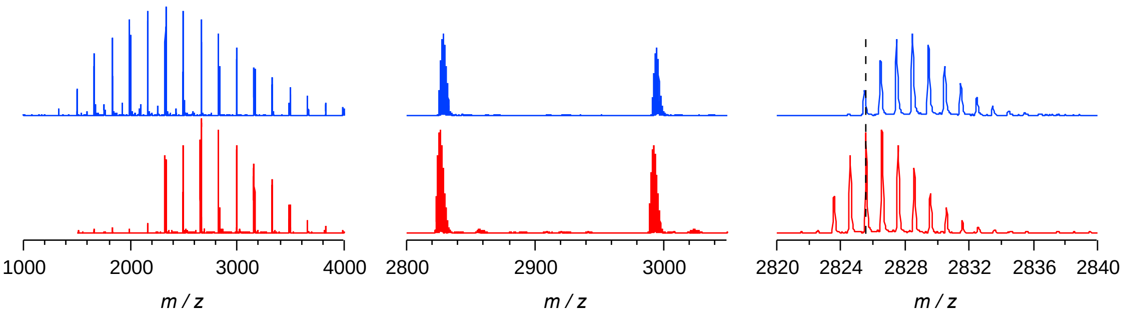

Figure S6. Full and expanded MALDI-TOF MS spectra of $\mathbf{L_{14}}$ (blue) and $\mathbf{C_{14}}$ (red).

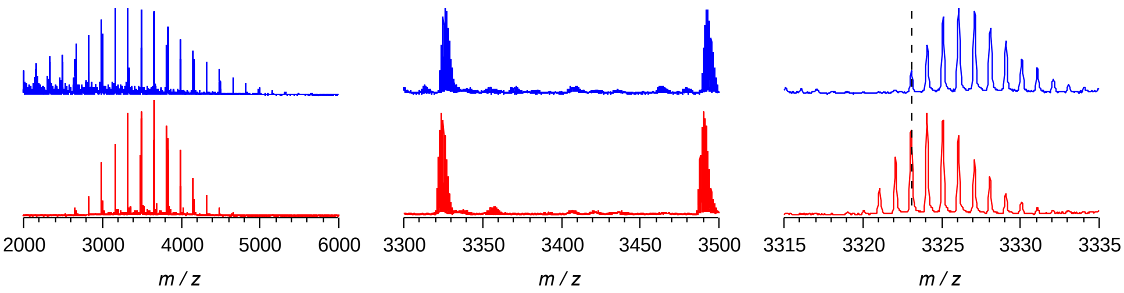

Figure S7. Full and expanded MALDI-TOF MS spectra of $\mathbf{L_{21}}$ (blue) and $\mathbf{C_{21}}$ (red).

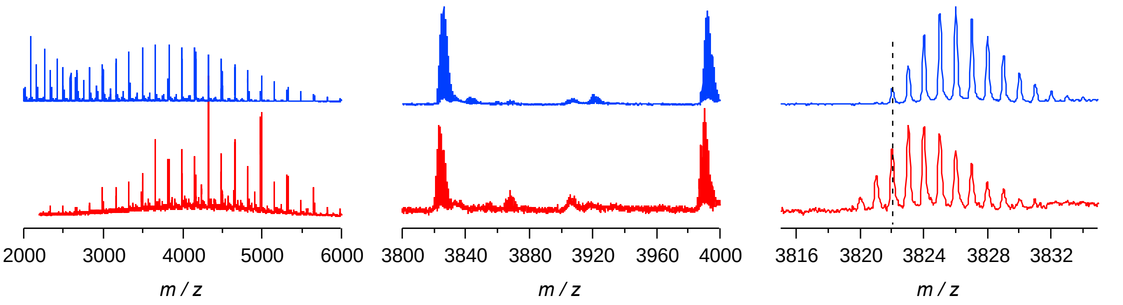

Figure S8. Full and expanded MALDI-TOF MS spectra of $\mathbf{L_{26}}$ (blue) and $\mathbf{C_{26}}$ (red).

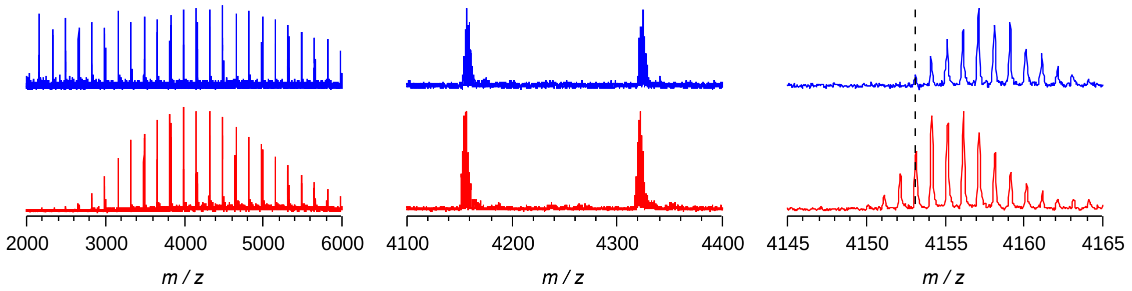

Figure S9. Full and expanded MALDI-TOF MS spectra of $\mathbf{L_{29}}$ (blue) and $\mathbf{C_{29}}$ (red).

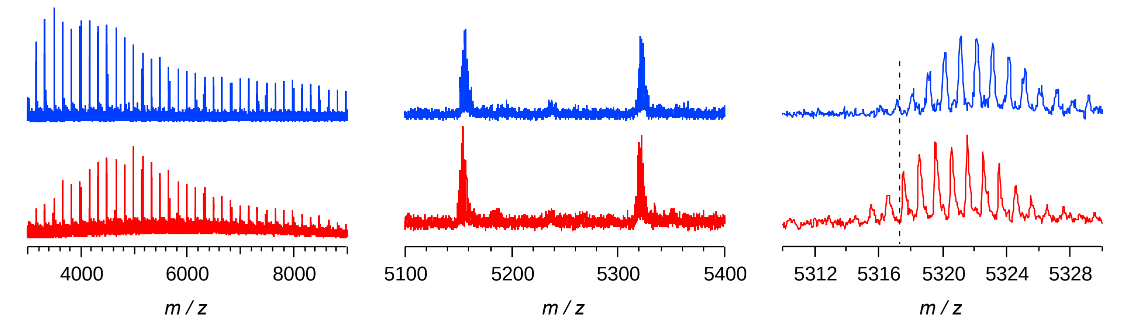

Figure S10. Full and expanded MALDI-TOF MS spectra of $\mathbf{L_{43}}$ (blue) and $\mathbf{C_{43}}$ (red).

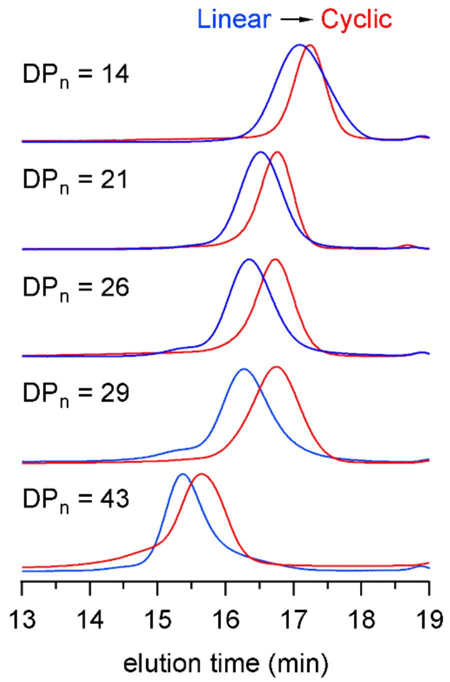

Figure S11. Analytical SEC traces of linear (blue) and cyclic (red) P3HT.

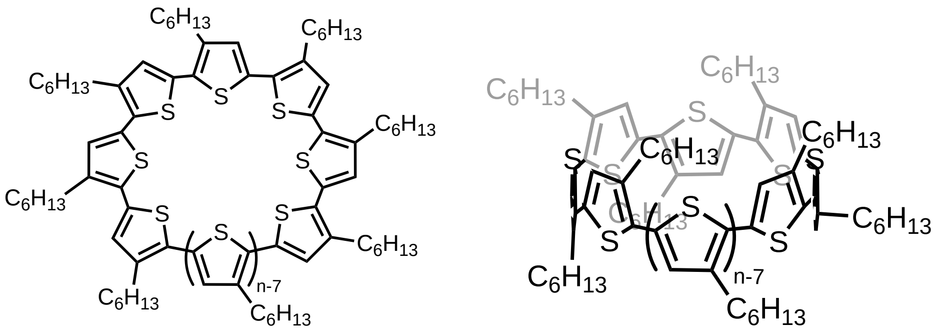

Figure S12 Cisoid (left) and transoid (right) structures of cyclic P3HT.

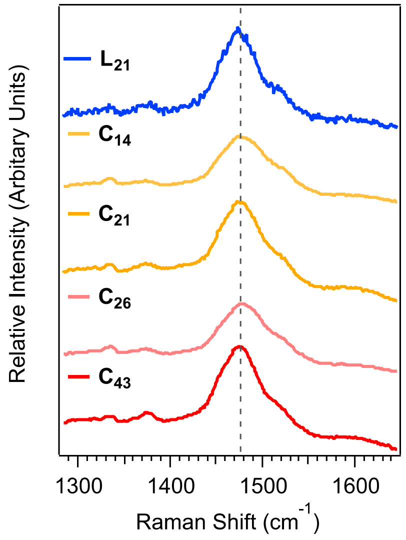

Figure S13. Raman spectra of linear and cyclic P3HT (**L_{21}**, **C_{14}**, **C_{21}**, **C_{26}**, **C_{43}**).

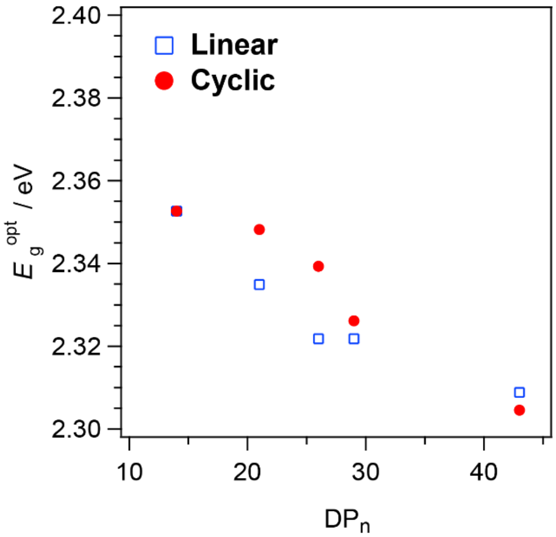

Figure S14. Optical band gaps (E_g^{opt}) of linear (blue square) and cyclic (red circle) P3HT calculated from the $S_0 \rightarrow S_2$ absorption band.

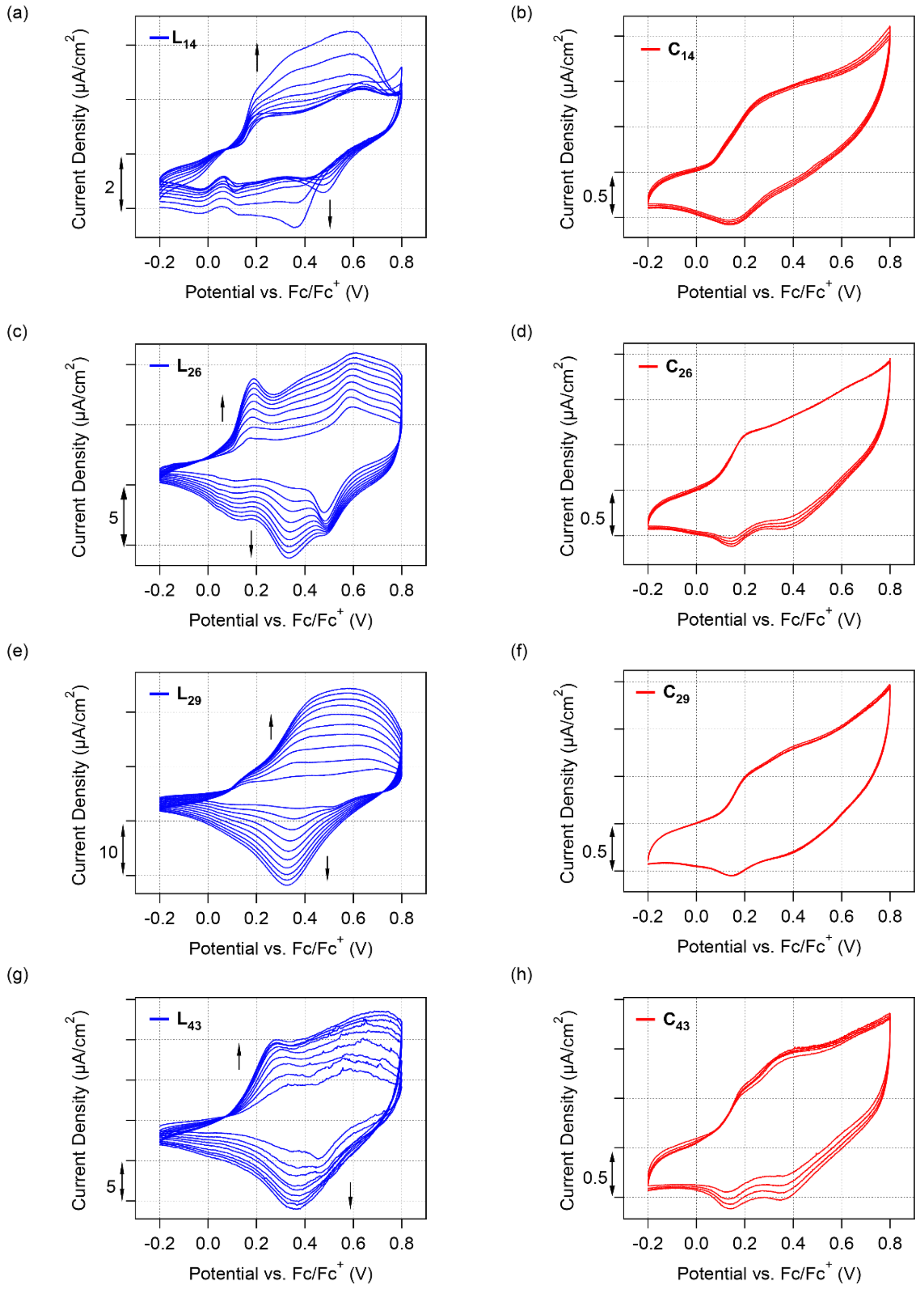

Figure S15. Cyclic voltammograms of (a) $\mathbf{L_{14}}$, (b) $\mathbf{C_{14}}$, (c) $\mathbf{L_{26}}$, (d) $\mathbf{C_{26}}$, (e) $\mathbf{L_{29}}$, (f) $\mathbf{C_{29}}$, (g) $\mathbf{L_{43}}$, and (h) $\mathbf{C_{43}}$ (Linear P3HT, 2nd–10th cycles; Cyclic P3HT, 2nd–5th cycles). For $\mathbf{C_{26}}$ and $\mathbf{C_{43}}$, the current density slightly increased due to the residual linear impurities.

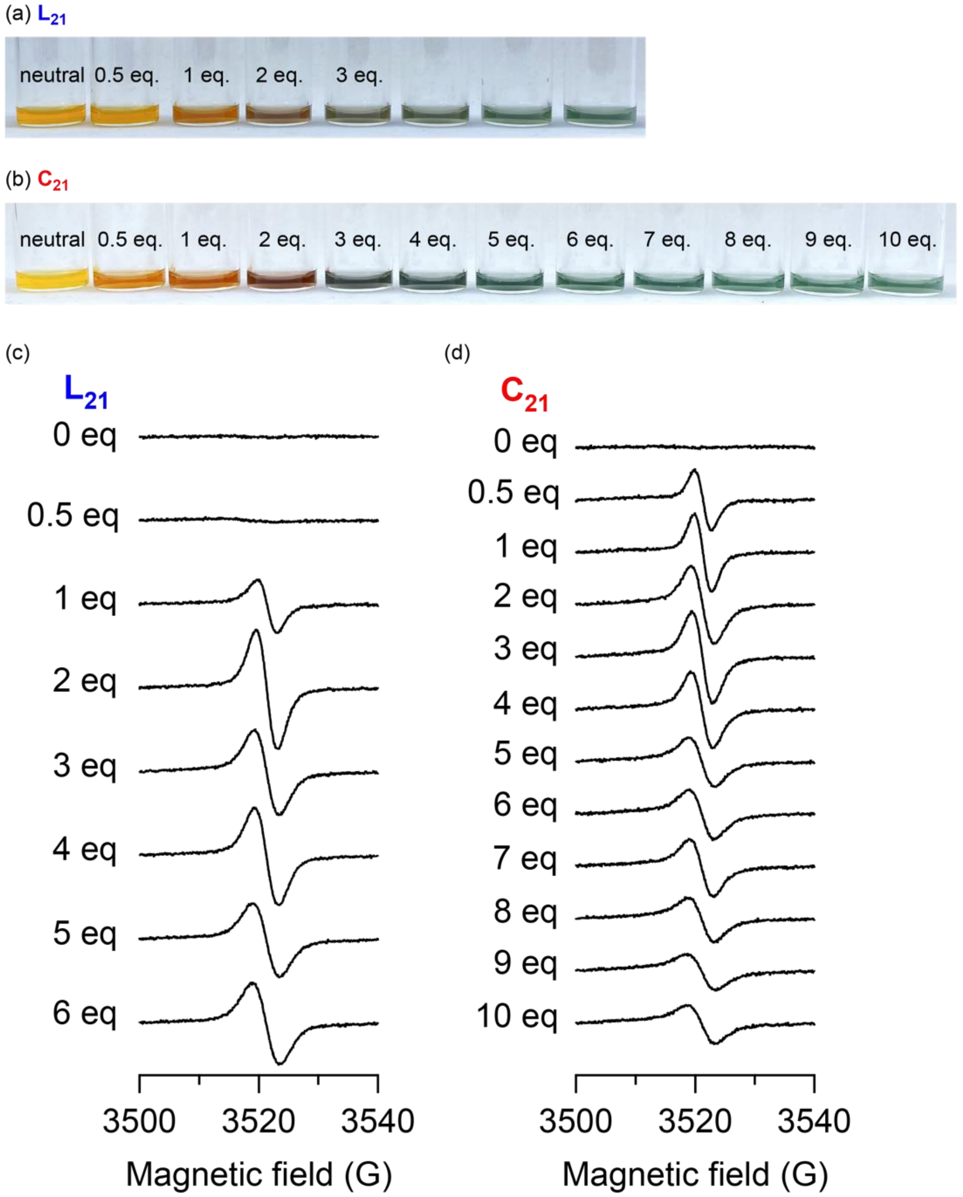

Figure S16. Photographs of (a) $\mathbf{L_{21}}$ and (b) $\mathbf{C_{21}}$ upon oxidation by $AgSbF_6$. ESR spectra of (c) $\mathbf{L_{21}}$ and (d) $\mathbf{C_{21}}$ upon oxidation. "eq" indicates the molar equivalent of added $AgSbF_6$ with respect to the P3HT macromolecules.

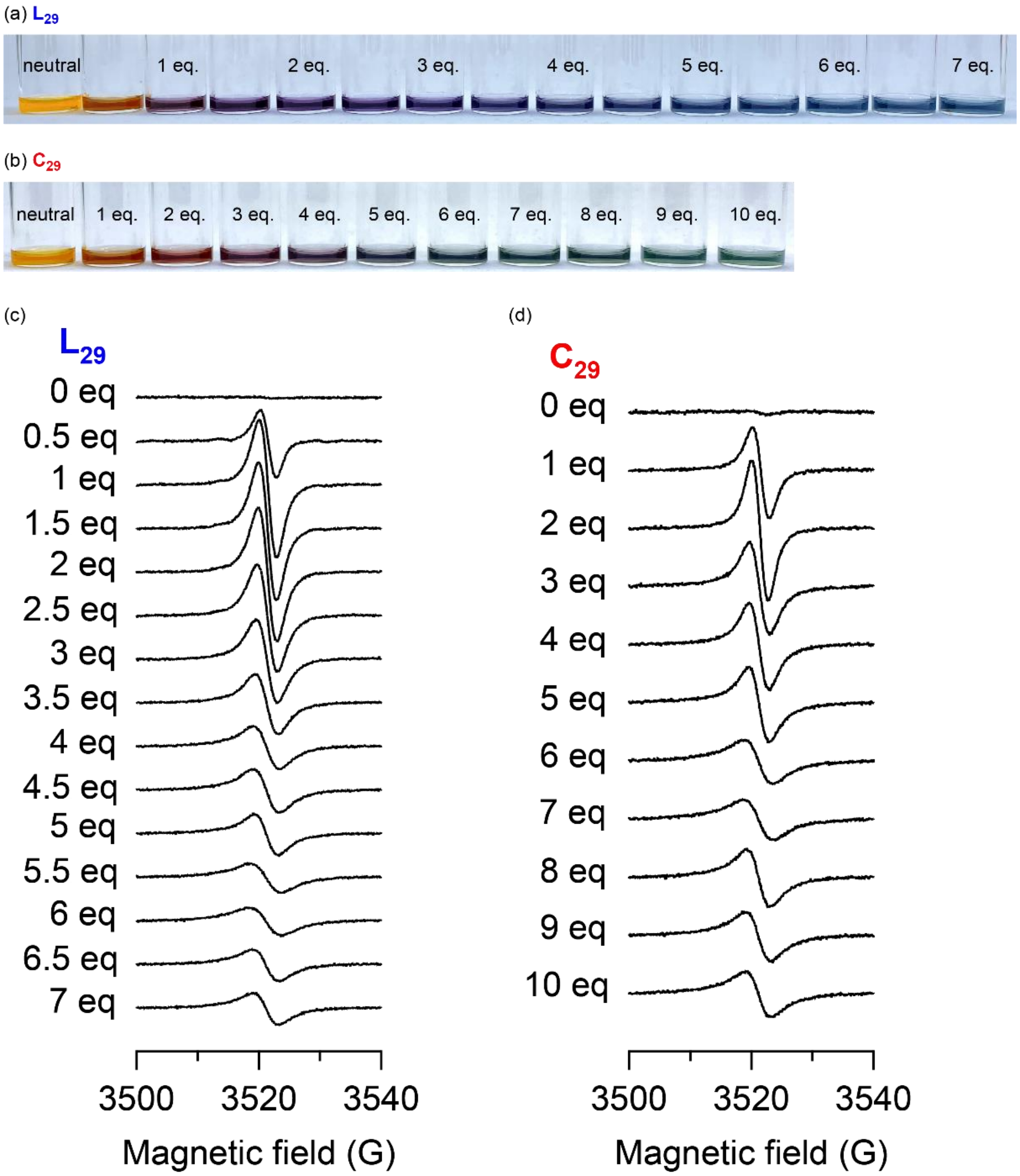

Figure S17. Photographs of (a) $\mathbf{L_{29}}$ and (b) $\mathbf{C_{29}}$ upon oxidation by $AgSbF_6$. ESR spectra of (c) $\mathbf{L_{29}}$ and (d) $\mathbf{C_{29}}$ upon oxidation. “eq” indicates the molar equivalent of added $AgSbF_6$ with respect to the P3HT macromolecules.

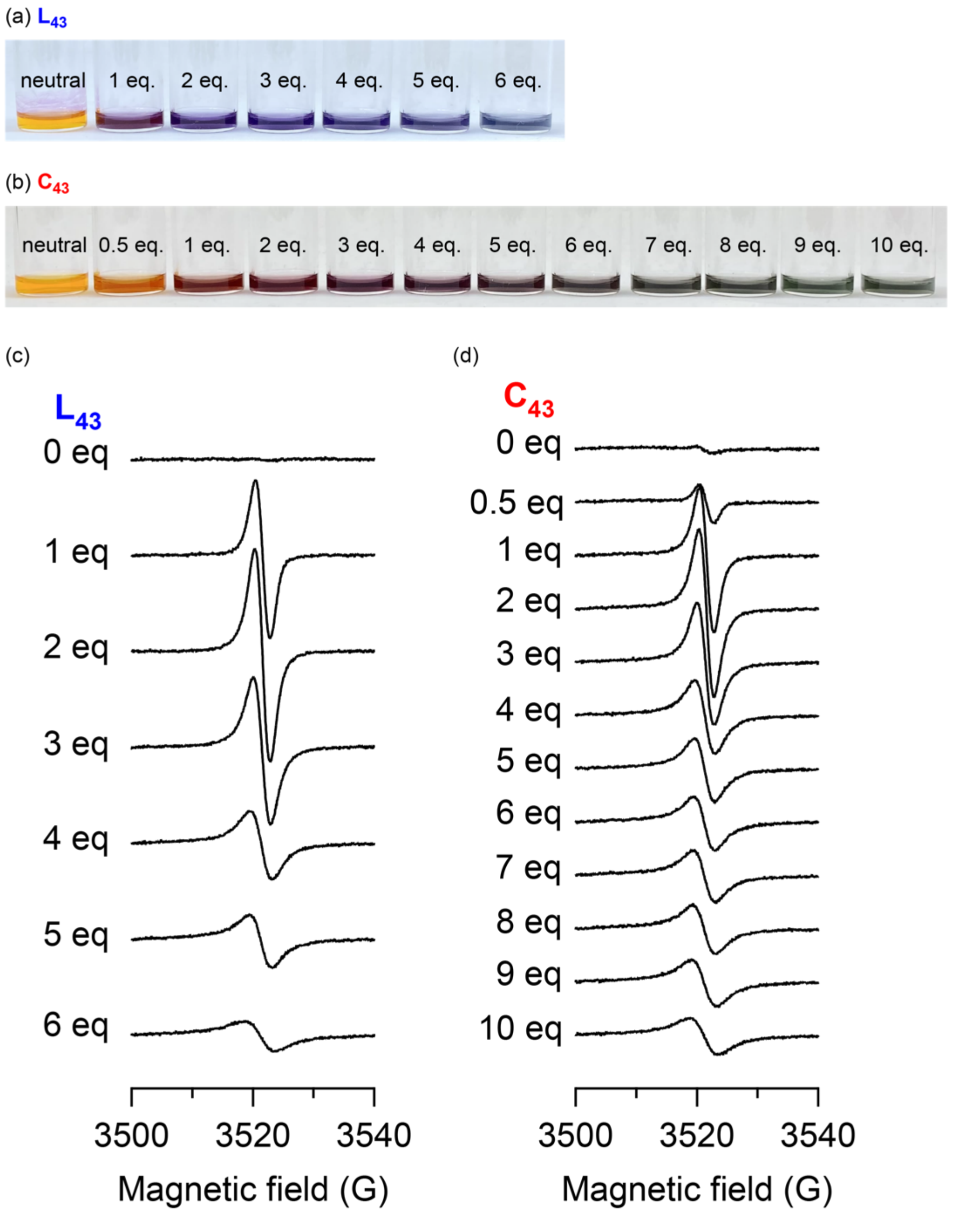

Figure S18. Photographs of (a) $\mathbf{L_{43}}$ and (b) $\mathbf{C_{43}}$ upon oxidation by $AgSbF_6$. ESR spectra of (c) $\mathbf{L_{43}}$ and (d) $\mathbf{C_{43}}$ upon oxidation. "eq" indicates the molar equivalent of added $AgSbF_6$ with respect to the P3HT macromolecules.

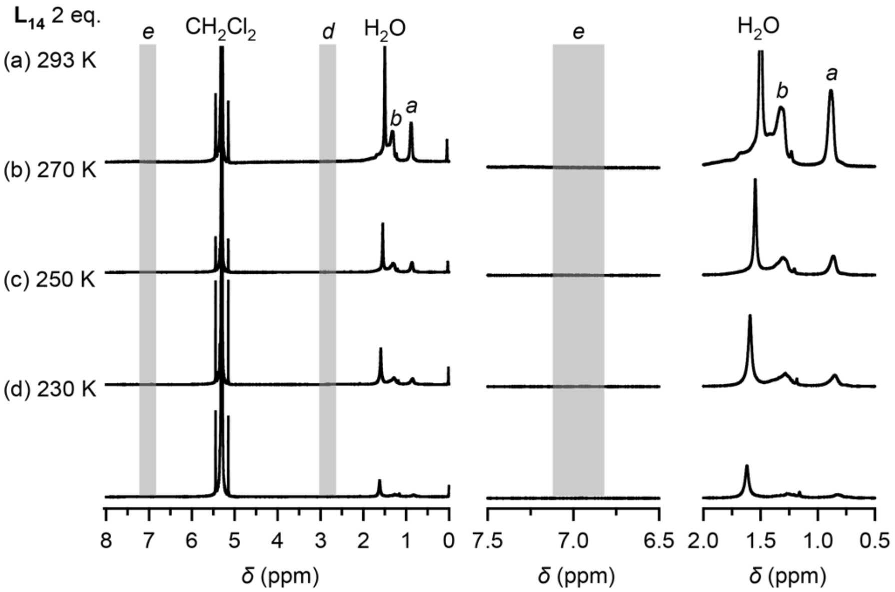

Figure S19. VT ^{1}H NMR spectra of $\mathbf{L_{14}}$ with 2 equiv of $AgSbF_6$.

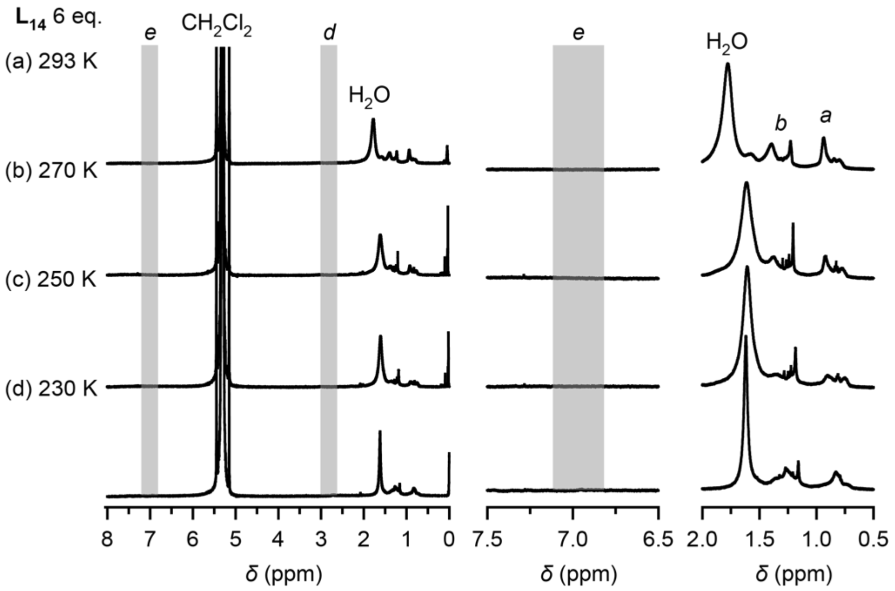

Figure S20. VT ^{1}H NMR spectra of $\mathbf{L_{14}}$ with 6 equiv of $AgSbF_6$.

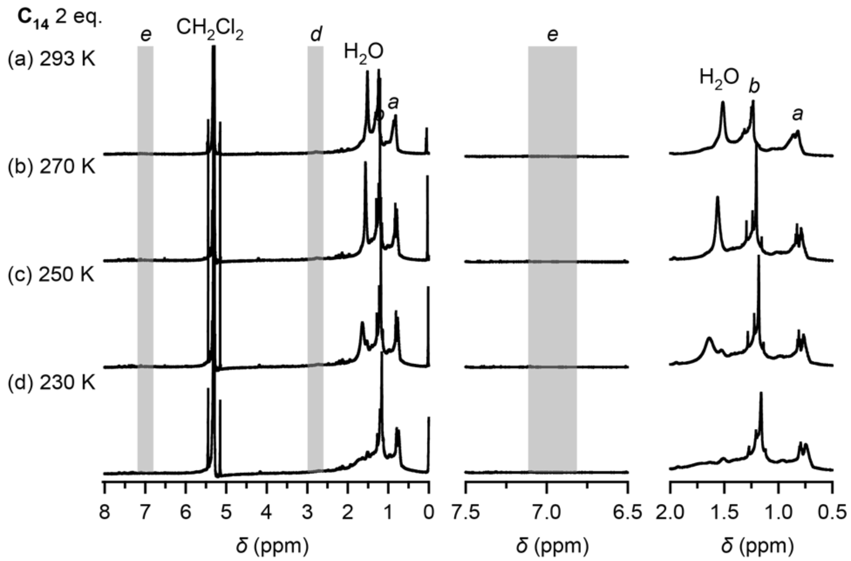

Figure S21. VT ^{1}H NMR spectra of $\mathbf{C_{14}}$ with 2 equiv of $AgSbF_6$.

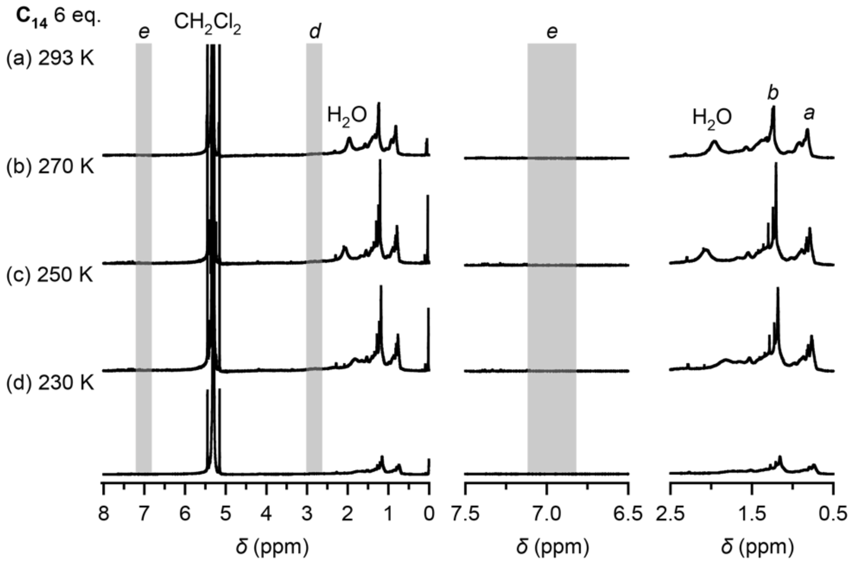

Figure S22. VT ^{1}H NMR spectra of $\mathbf{C_{14}}$ with 6 equiv of $AgSbF_6$.
